# Supplementary material for: Evaluation of Histone Demethylase Inhibitor ML324 and Acyclovir against Cyprinid herpesvirus 3 Infection
Source: Viruses. 2023 Jan 5;15(1):163. doi: 10.3390/v15010163 (PMC9863241; doi:10.3390/v15010163)
Supplement: Supplementary file 1 [file viruses-15-00163-s001.zip › viruses-2070919-supplementary.pdf]

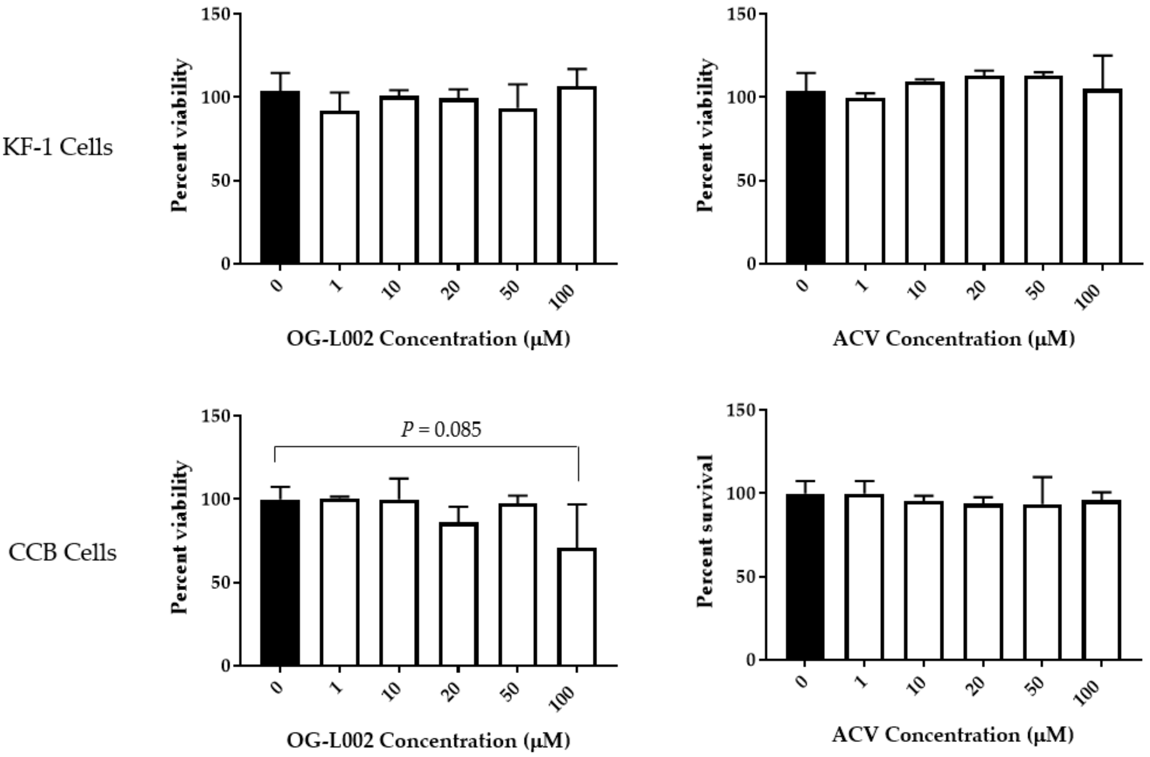

Supplemental Figure S1. The cytotoxicity of OG-L002 and ACV in vitro. KF-1 cells (top panel) or CCB cells (bottom panel) were incubated with the indicated concentration of OG-L002 or ACV for 24 h. The treatment was then removed, and the cells were washed once with PBS and further incubated for 24 h in tissue culture media (DMEM) with 5% serum and antibiotics as described in the materials and methods. Cell viability was evaluated with the XTT cell viability kit III and expressed as a percentage of the mock-treated control (n = 3). The statistical difference between the DMSO-treated control (0) and 100 ▯MOG-L002 is marked above the line with a Bonferroni-corrected p-value ($p > 0.05$, which is not significant) calculated using a two-way ANOVA.
